# Activated phosphoinositide 3-kinase delta syndrome 1 and 2 (APDS 1 and APDS 2): similarities and differences based on clinical presentation in two boys

**DOI:** 10.1186/s13223-020-00420-6

**Published:** 2020-04-01

**Authors:** Marlena Ewertowska, Elżbieta Grześk, Anna Urbańczyk, Anna Dąbrowska, Katarzyna Bąbol-Pokora, Monika Łęcka, Sylwia Kołtan

**Affiliations:** 1 Faculty of Medicine, Collegium Medicum in Bydgoszcz, Nicolaus Copernicus University in Toruń, Jagiellońska 13, 85-067 Bydgoszcz, Poland; 2 Department of Paediatrics, Hematology and Oncology, Collegium Medicum in Bydgoszcz, Nicolaus Copernicus University in Toruń, Curie Skłodowskiej 9, 85-092 Bydgoszcz, Poland; 3 Department of Paediatrics, Oncology, Hematology and Diabetology, Medical University of Łódź, Al. Kościuszki 4, 90-419 Łódź, Poland

**Keywords:** PI3Kδ, Primary immunodeficiency, APDS, Hyper IgM, Growth hormone, Genetic testing

## Abstract

**Background:**

Activated PI3K delta syndrome (APDS) belongs to the heterogeneous group of primary immunodeficiency disorders (PIDs). Progress in next-generation sequencing (NGS) enabled identification of gain-of-function mutations in phosphoinositide 3-kinase (PI3K) genes. Depending on the type of causative mutation, APDS is classified into two types: APDS 1 and APDS 2. To date, less than 100 cases of APDS have been reported. Clinical symptoms of APDS result from impaired immune regulation and are clinically manifested by recurrent infections, allergies, lymphoproliferation and autoimmunity. They show similarity to other PIDs. Therefore, many patients were diagnosed incorrectly. The availability of genetic testing has allowed establishing the correct diagnosis in increasing number of patients suffering from APDS.

**Case presentations:**

The first male patient presented in infancy with recurrent infections. Subsequently he was found to suffer from hepatosplenomegaly, early portal hypertension, massive lymphoproliferation and hypogammaglobulinemia. The common E1021K mutation in the PI3KCD gene was identified. The patient underwent successful hematopoietic stem cell transplantation with resolution of most symptoms. The second patient suffered from persistent growth retardation since early life, facial dysmorphism and recurrent respiratory infections from early childhood. He was found to have systemic lympho-proliferation, panhypoglobulinemia and impaired antibody responses to vaccines. The introduction of NGS in Poland enabled rapid identification of a mutation in the PI3KR1 gene. Growth hormone administration seemed to have worsened the lymphoproliferation.

**Conclusions:**

Patients with suspected common variable immunodeficiency (CVID) and additional symptoms, such as allergy, facial dysmorphia, short stature, enhanced lymphoproliferation and lack of adequate response to human immunoglobulin replacement therapy, should be considered for NGS-based genetic testing. It may substantially shorten the time needed to establish the correct diagnosis, direct appropriate treatment and avoid potentially harmful therapies. To date, few cases of APDS have been described. It is important to report each of them to establish clinical indices and laboratory biomarkers of APDS 1 and APDS 2, to develop the standards of care in these conditions.

## Background

Primary immunodeficiency disorders (PIDs) are a group of immune system diseases, many of them caused by genetic defects [1, 2].

Phosphoinositide 3-kinases (PI3Ks) are the family of enzymes expressed in various classes of cells and involved in signal transduction via a few main pathways. Class I PI3Ks are expressed in leukocytes and catalyze the synthesis of a second messenger, phosphatidylinositol 3,4,5-triphosphate (PIP3). PI3Ks are heterodimers composed of a catalytic and regulatory unit [3, 4].

APDS is a consequence of a gain-of-function (activating) mutation. APDS was first described in 2014 in a small group of patients with PID of unknown etiology [1, 5]. Using next-generation sequencing (NGS), a rare heterozygous mutation of the *PI3KCD* gene encoding catalytic subunit of the PI3K (PI3Kδ) has been identified. Then, other mutations of the *PI3KR1* gene encoding the regulatory subunit (p85α PI3Kδ) have been described. Depending on the literature source, between 47 and 100 new cases of APDS have been reported since then [1, 3]. However, the exact number of patients with this condition is unknown. APDS resulting from mutation of the *PI3KCD* has been classified as APDS 1, whereas the disease associated with the mutation of the *PI3KR1* is referred to as APDS 2 [5].

APDS is a complex deficiency of cellular and humoral immunity, which explains a heterogeneity of its clinical manifestations. Most patients present with recurrent respiratory infections, such as otitis media, sinusitis and pneumonia (typically with *Haemophilus influenzae* and *Streptococcus pneumoniae* as etiological factors), which suggests the presence of an antibody deficiency [1, 3, 6]. Also, viral infections, with herpesvirus simplex (HPV), cytomegalovirus (CMV) and Epstein–Barr virus (EBV) are reported, as well as parasitic and fungal infections, suggesting an impairment of T cell function [1, 3]. According to literature, patients with APDS 1 may also present with gastrointestinal infections [6, 7]. Another clinical manifestation are local and systemic lymphoproliferative disorders and hepatosplenomegaly observed since early childhood [3, 7–9]. Abnormalities in laboratory results include immune cytopenia and altered distribution of lymphocyte subpopulations [1, 5, 7, 8]. Because of the tendency to lymphoproliferation, patients with APDS are at increased risk of neoplastic transformation, mainly to hematologic malignancies [1, 3, 9]. APDS 1 and APDS 2 show some differences in their clinical presentation. Similarities and differences between these two disease entities are summarized in Table 1.

**Table 1:** Clinical characteristics of APDS 1 and APDS 2 reported in the literature and manifestations of these conditions in our patients [1, 3, 5, 7, 11]

Manifestation	APDS 1		APDS 2	
	Typical symptoms	Symptoms in the patient 1	Typical symptoms	Symptoms in the patient 2
Infectious complications				
Recurrent respiratory infections				
Pneumonia	+	+	+	+
Bronchitis	+	+	+	+
Sinusitis	+	+	+	+
Otitis	+	−	+	+
Viral infections				
HPV	+	−	+	−
CMV	+	+	+	−
EBV	+	−	+	−
Adenovirus	+	+	−	−
Parasitic and fungal infections	+	+	+	−
Gastrointestinal infections	+	+	−	−
Non-infectious complications				
Lymphadenopathy	+	+	+	+
Splenomegaly	+	+	+	−
Hepatomegaly	+	+	+	−
Nodular lymphoid hyperplasia in the gastrointestinal tract	+	+	+	−
Signs of facial dysmorphia	−	−	+	+
Short stature	−	−	+	+
Intellectual disability	−	−	+	−
Microcephalia	−	−	+	−
Mutation				
E1021K	+	+	−	−
N334K	+	−	−	−
E525K	+	−	−	−
C416R	+	−	−	−
NM_181523	−	−	+	+
Laboratory abnormalities				
Lower concentrations of IgG and IgA	+	+	+	+
Elevated concentration of IgM	+	+	+	−
Lymphopenia CD19+	+	+	+	+
Elevated count of T lymphocytes CD8+	+	+	+	−
Inverted CD4/CD8 ratio	+	+	+	+
Lower count of naïve T lymphocytes CD4+ (CD4+CD45RA+)	+	+	+	−
Lower count of naïve T lymphocytes CD8+ (CD8+CD45RA+)	−	−	+	−
Treatment				
Immunoglobulin replacement therapy	+	+	+	+
Immunosuppressive therapy	+	−	+	−
Allo-HSCT	+	+	+	−

In this paper, we present two boys who had been diagnosed with APDS 1 and APDS 2, respectively. In the first case, the suspicion of aPID has been raised already in early childhood. However, because of limited access to appropriate diagnostic (in particular, genetic) tests, the diagnosis was established only after a few years. In the second case, clinical manifestations suggestive of an immunodeficiency disorder also emerged during early childhood, but PID was considered as a diagnosis only after the end of the first decade of life. As NGS-based testing has been already available in Poland, it took only a few months to establish the final diagnosis.

## Case presentation

### Case 1

The 13-year-old boy was born at term from an uncomplicated pregnancy (1st pregnancy, 1st delivery). The patient was delivered via cesarean due to the risk of fetal distress, in good general status, with a birthweight of 3900 g and body length of 57 cm. In the family history there was pollen and foot allergy in maternal uncle. Neonatal period was complicated by congenital pneumonia. Beginning at 6 months of age, the boy frequently suffered from recurrent chronic lower respiratory infections. The diagnostic process carried out in an outpatient setting, excluded cystic fibrosis and diagnosed with asthma. Since the neonatal period, the patient presented with periodically exacerbating diarrhea, with a few bowel movements per day. During one episode of the diarrhea exacerbation, *Staphylococcus aureus* was isolated from the stool culture. Gastrointestinal infection was diagnosed and antibiotic therapy was used. Because of a suspected immune disorder, the boy at the age of 3 was referred to the Department of Pediatrics, Hematology and Oncology for immunological diagnostics. Physical examination demonstrated pallor of the skin, hypertrophic palatine tonsils (type 4 in Pirquet classification), hepatomegaly (the largest dimension was 13 cm) and splenomegaly (the largest dimension was 11 cm) (Table 1). Abnormalities in laboratory results included hypochromic anemia with a normal concentration of ferritin, hypogammaglobulinemia, a titer of anti-HBs antibodies were 1.0 mIU/ml, a titer of CMV-IgG was < 0.2 IU/ml and concentration of isohemagglutinins was 1. Analysis of lymphocyte subpopulations showed a decrease in the percentages and absolute counts of B lymphocytes and T-helper cells, along with an increase in the proportion and absolute count of T-cytotoxic lymphocytes. The population of T-helper lymphocytes contained smaller than normal percentage of naive cells and normal proportion of memory cells, whereas a slight increase in the proportion of naive cells and considerably elevated percentage of memory cells was observed in the population of T-cytotoxic lymphocytes, which could reflect the frequent infections that the patient had [1]. The results of laboratory tests are summarized in Table 2. The boy was qualified for immunoglobulin replacement therapy. Because of concomitant anemia and hepatosplenomegaly, doppler flow in liver vessels were examined to exclude potential thromboembolism. Normal flow through the portal vein was observed at a speed of 22–25 cm/s. Moreover, the patient underwent gastroscopy, which revealed the signs of chronic gastro-duodenitis and dilated venous vessels of the fundus and significant unevenness of the gastric and duodenal mucosa, suggestive of early portal hypertension. Furthermore, *Helicobacter pylori* infection was diagnosed based on histopathological examination of gastrointestinal biopsy specimens.

**Table 2:** Results of laboratory tests in the hereby reported patients

Laboratory parameter	Case 1^b^	Age-specific norm^a^	Case 2^c^	Age-specific norm^a^
Leukocyte count	5.14 K/µl	5–15 K/µl	6.48 K/µl	5–13 K/µl
Neutrophil count	3.23 K/µl	1.5–8 K/µl	3.04 K/µl	2–8 K/µl
Hemoglobin concentration	8.8 g/dl ↓↓	11.5–14.0 g/dl	13.0 g/dl	11.5–15.5 g/dl
Platelet count	145 K/µl	100–490 K/µl	373 K/µl	100–450 K/µl
IgG concentration	<1.56 g/l ↓↓↓	4.28–12.3 g/l	2.0 g/l ↓↓↓	8.5–13.0 g/l
IgA concentration	0.33 g/l ↓↓	1.08–2.43 g/l	<0.06 g/l ↓↓↓	0.91–2.55 g/l
IgM concentration	2.3 g/l	0.3–1.12 g/l	0.27 g/l ↓↓↓	0.66–1.55 g/l
B lymphocytes (CD 19+)	63/µl (4.72%) ↓↓↓	400–1700/µl	164/µl (5.4%) ↓↓	200–600/µl
T lymphocytes (CD 3+)	1515/µl (78.9%) ↓↓	2120–2830/µl	2202/µl (72.5%)	800–3500/µl
T-helper lymphocytes (CD3+CD4+)	207/µl (15.32%) ↓↓	640–1560/µl	777/µl (25.5%)	400–2100/µl
T-helper memory lymphocytes (CD3+CD4+CD45RO+)	13.88% (186/µl)	8.7–25.9%	4.5% (137/µl) ↓↓↓	27.2–62.0%
Naïve T-helper lymphocytes (CD3+CD4+CD45RA+)	2.35% (31/µl) ↓↓↓	13.3–37.8%	61.1% (1861/µl)	31.1–66.3%
T-cytotoxic lymphocytes (CD3+CD8+)	680/µl (50.47%) ↑	200–630/µl	1075/µl (35.3%)	200–1200/µl
T-cytotoxic memory lymphocytes (CD3+CD8+CD45RO+)	46.18% (622/µl) ↑↑	3.9–20.2%	54.8% (1669/µl) ↑↑	15.9–46.4%
Naïve T-cytotoxic lymphocytes (CD3+CD8+CD45RA+)	24.9% (336/µl) ↑	6.9–22%	16.6% (506/µl) ↓↓	44.1–77.1%

^a^Differences in normal ranges reflect different age of patients at the time of laboratory testing

^b^Laboratory test was performed on the patient aged 3 years

^c^Laboratory test was performed on the patient aged 10 years

**Fig. 1:**
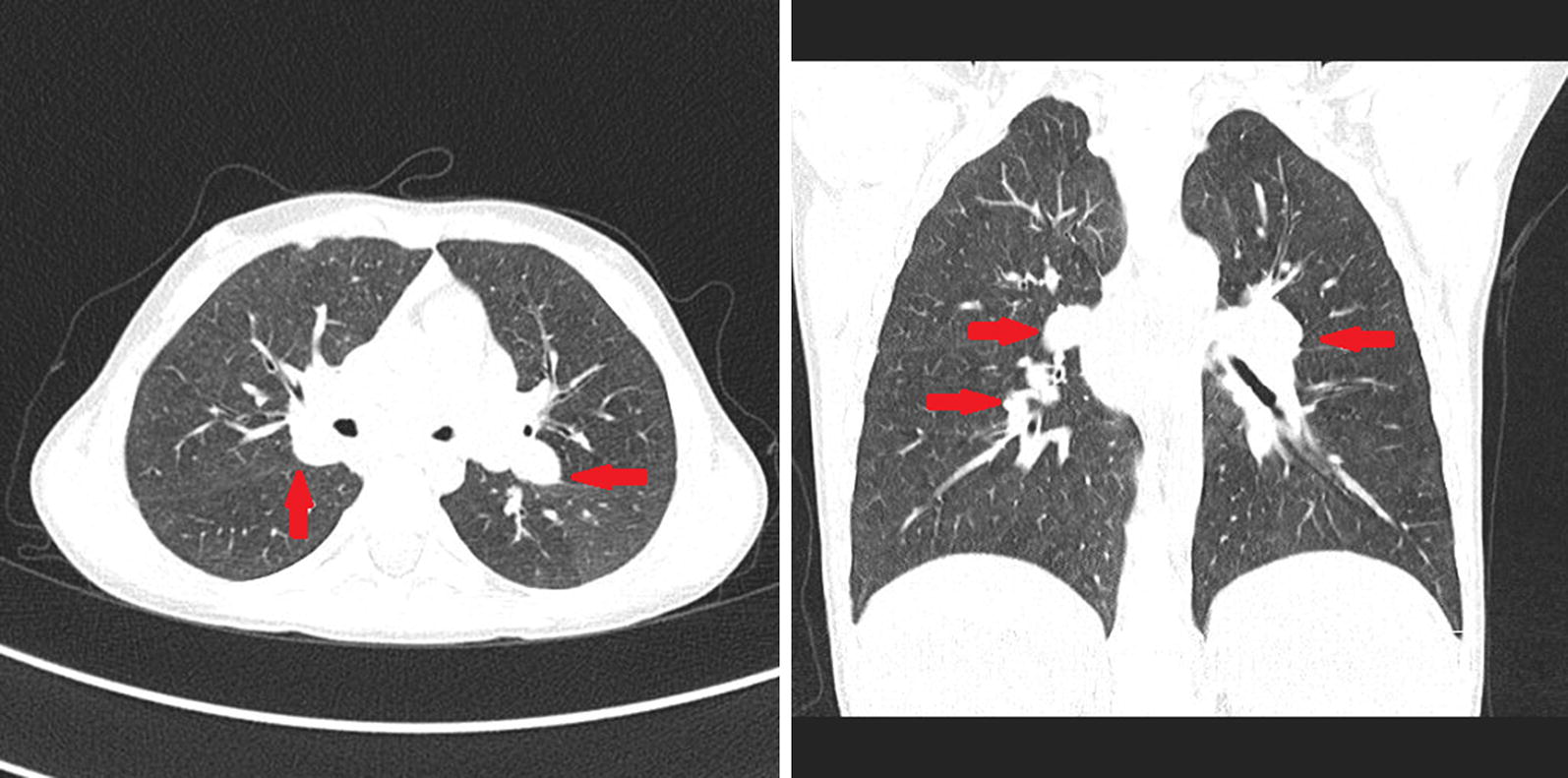
High-resolution computed tomography scan documenting severe lymphoproliferation within mediastinal lymph nodes of the 8-years-old boy with APDS 1

The clinical presentation raised suspicion of congenital immunodeficiency disorder. Differential diagnoses included hyper IgM syndrome, X-linked lymphoproliferative disease (XLP) and CVID, but the true cause of ailments has not been established at this timepoint. During subsequent years, the boy experienced massive hemorrhagic esophagitis with fungal superinfection, was diagnosed with chronic colorectal inflammation, chronic sinusitis and bronchitis, and showed the signs of gradually progressing lymphoproliferation (Fig. 1). Six years after the initial referral, the patient was eventually diagnosed with APDS 1 based on the result of NGS carried out abroad. The E1012K mutation was found in the PI3KCD [7]. The patient’s family has not been tested for the above mutation. Because of the unfavorable phenotype of the disease (massive lymphoproliferation with the risk of neoplastic transformation, respiratory infections resistant to immunoglobulin replacement therapy, adenoviral infections, cytomegaly and poor overall quality of life), the boy was qualified for allogenic hematopoietic stem cell transplantation (allo-HSCT) from his histocompatible healthy brother. In line with the EBMT/ESID guidelines for hematopoietic cell transplantation for primary immunodeficiencies, a less toxic conditioning regimen was used, with intravenous busulfan (2.8 mg/kg daily for 4 days), fludarabine (45 mg/m^2^ daily for 4 days) and anti-lymphocyte globulin (8 mg/kg per 3 days for 4 days). Currently, 5 years after the allo-HSCT, the boy presents with full donor chimerism, shows no signs of active graft versus host reaction and complete immunological reconstitution. The pathological non-neoplastic lymphoproliferation has resolved completely, and the boy does not require immunoglobulin replacement therapy. After the allo-HSCT, the patient underwent complete revaccination. The only health problem present in the boy currently is chronic paranasal sinusitis.

### Case 2

The second patient was an 11-year-old boy with a remarkable family history (mother after anti-lymphoma treatment and nephrectomy). The boy was born at term from an uncomplicated pregnancy (4th pregnancy, 2nd delivery). The patient was delivered via cesarean due to the post-nephrectomy status of his mother, in good general status, with a birthweight of 3300 g and body length of 55 cm. During the neonatal period, the boy presented with muscle tone abnormalities, which resolved after rehabilitation. Since the beginning of this life, the patient showed the signs of a growth disorder; differential diagnoses included abnormal thyroid function, hypoparathyroidism, adrenal insufficiency, celiac disease and Silver-Russel syndrome, but all these conditions were eventually excluded. Beginning in early childhood, the boy suffered from recurrent respiratory infections, primarily bronchitis and pneumonia. At 9 years of age, the patient experienced multiple episodes of recurrent upper respiratory infections, including sinusitis and otitis media, along with a few episodes of bronchitis. Analysis of immunoglobulin levels conducted in an outpatient setting revealed extremely low concentrations of IgA, IgM and IgG. The boy was referred to the Department of Pediatrics, Hematology and Oncology for further immunological diagnostics. Physical examination demonstrated deficits of body weight and height (below the 3rd percentile for age and sex), signs of facial dysmorphia (triangular face with disproportionally large neurocranium, hypertelorism, downward slanting eyes, broad nasal root, low set ears), geographic tongue and prominent papillae of the tongue’s base. Analysis of lymphocyte subpopulations demonstrated a decrease in the percentage and absolute count of B cells, with profound disorders in the distribution of CD3+CD4+CD45RO+ (memory) lymphocytes (decreased proportion with normal proportion of naïve, CD3+CD4+CD45RA+cells) and T lymphocytes CD8+ (a significant increase in the proportion of memory cells, with a decrease in the proportion of naïve cells) (Table 2). Testing of humoral immunity confirmed complete inability to synthesize specific antibodies. Ultrasonography of lymph nodes showed systemic lymphoproliferation with a reactive phenotype. The results raised suspicion of a CVID. Another potential diagnosis was a genetic syndrome associated with PID, the deficit of growth and facial dysmorphia. The patient was qualified for NGS-based testing, which demonstrated a mutation in the *PI3KR1* gene (NM_181523.2); the result was later confirmed by means of direct sequencing. The patient’s family has not been tested for the above mutation. The final diagnosis of APDS 2 was established 9 months after an initial suspicion of an immunodeficiency disorder has been raised.

Currently, the boy is on subcutaneous immunoglobulin replacement therapy, which contributed to a significant reduction of the incidence of opportunistic infections and a decrease in lymphoproliferation severity. Since the patient presented with height deficit, his parents arranged and funded growth hormone therapy at a private clinic despite a written opinion from the immunologist about questionable effectiveness of such treatment in a child with APDS 2 [10]. The treatment was discontinued after a year: the boy gained 2 cm in height, but the implementation of the hormonal therapy co-existed with a substantial exacerbation of the systemic lymphoproliferative process. Furthermore, hyperplastic lingual papillae caused difficulty breathing that led to acute respiratory failure. The boy required Intensive Care Unit stay, intubation and respiratory support. The hyperplastic papillae of the tongue’s base had to be removed surgically. Histopathological examination of the surgical specimens confirmed massive benign lymphocytic infiltration. After discontinuation of the growth hormone therapy, the size of lymph nodes in all locations decreased considerably, and according to the boy’s parents, his quality of life returned to the pretreatment level (Table 1).

## Discussion and conclusions

The growing availability of NGS-based testing facilitated detection of rare monogenic disorders. Both our patients presented with recurrent respiratory infections and lymphoproliferation. The boy with APDS 1 also had a history of gastrointestinal infections of both viral and bacterial etiology and hepatosplenomegaly. According to sparse published reports, patients with APDS 2 may present with growth deficits, facial dysmorphia, intellectual disability and microcephalia [1, 3, 10]. These clinical features can be used to distinguish APDS 2 from APDS 1, as to this date, they have not been reported in patients with the latter condition. Our patient with APDS 2 presented with facial dysmorphia and growth deficit, which is consistent with the previously published reports [3, 10]. A laboratory finding in APDS patients is impaired antibody production, primarily the deficiency of immunoglobulins G and A, with a concomitant increase in IgM level. While the first of our patients showed this pattern of alterations in immunoglobulin levels, the boy with APDS 2 presented with a severe deficiency of immunoglobulins in all main classes. Other laboratory abnormalities observed in APDS include a decrease in absolute count of B lymphocytes and naïve T lymphocytes, both cytotoxic (CD3+CD4+) and helper (CD3+CD8+) cells [1, 3, 11].

Most patients with APDS reported in literature received human immunoglobulin replacement therapy since early childhood to reduce the incidence of opportunistic infections [6, 7]. Some authors used rapamycin in patients with APDS 1 and APDS 2 and reported beneficial effects of the treatment in the form of lesser incidence of opportunistic infections, resolution of lymphoproliferative process and hepatosplenomegaly [6, 8]. Although long-term treatment with rapamycin may be highly beneficial in patients with APDS 2, it is also associated with the risk of adverse events outside the immune system [8]. In patients with severe APDS, especially those with enhanced lymphoproliferation and profound lymphocyte dysfunction, allo-HSCT seems to be a therapeutic option, considering the potential risk of neoplastic transformation [6, 8, 12, 13]. We have implemented immunoglobulin replacement therapy in both our patients. In the boy with APDS 1, the treatment did not contribute to a substantial improvement of clinical status. Conversely, despite the treatment, the patient presented with recurrent bacterial and viral infections and progressive lymphoproliferation and hepatosplenomegaly. Considering the severity of APDS 1, the patient eventually received allo-HSCT from a histocompatible family donor with a good clinical effect. This confirms the appropriateness of such an approach in patients with particularly severe APDS.

In the second patient, the boy with APDS 2, immunoglobulin replacement therapy contributed to a considerable decrease in the incidence of respiratory infections and resolution of lymphoproliferation. As the boy presented with a growth deficit, he also received growth hormone therapy. The treatment has been implemented against the immunologist’s suggestion based on sparse published evidence that APDS 2 may be associated with resistance to growth hormone [3, 10]. The therapy has been discontinued after a year due to its poor clinical effect and a substantial increase in lymphoproliferative activity, the onset of which co-existed with the implementation of the treatment.

Available evidence suggests that mutation of the *PI3KR1* gene may have an oncogenic potential. Somatic mutations of the *PI3KR1* have been found in patients with Burkitt lymphoma, endometrial cancer, colorectal cancer, ovarian cancer, gastric cancer and malignant melanoma, which points to likely oncogenic potential of this genetic defect [8]. Therefore, the presence of a severe lymphoproliferative process in our patient with APDS 2 raised an oncological alert. However, after discontinuation of growth hormone therapy, we observed a substantial decrease in lymph node size in all locations, which might confirm a link between lymphoproliferation and the hormonal treatment. Moreover, our experiences are consistent with a sparse published data on resistance to growth hormone in APDS 2 patients. Our observations suggest that treatment with this hormone is not only ineffective but may even pose a threat to the patient (respiratory failure).

To summarize, patients with suspected APDS should undergo genetic testing as it may substantially shorten the time needed to establish the correct diagnosis. Patients with suspected CVID and additional symptoms, such as intellectual disability, facial dysmorphia, allergy, short stature, enhanced lymphoproliferation and lack of adequate response to human immunoglobulin replacement therapy, should be qualified for NGS-based genetic testing. Unfortunately, the availability and financing of NGS in Poland is still limited. Due to these difficulties, future studies should define clinical indices and laboratory biomarkers of APDS 1 and APDS 2, to develop the standards of care in these conditions.

## Data Availability

Not applicable.

## Abbreviations

APDS: Activated phosphoinositide 3-kinase delta syndrome
PID: Primary immunodeficiency disorder
NGS: Next-generation sequencing
EBV: Epstein–Barr virus
CMV: Cytomegalovirus
HSV: Herpes simplex virus
VZV: Varicella zoster virus
XLP: X-linked lymphoproliferative disease
CVID: Common variable immunodeficiency
allo-HSCT: Allogenic hematopoietic stem cell transplantation
HRCT: High-resolution computed tomography
CID: Combinated immunodeficiency
